# Development and Characterization of Stingless Bee Propolis Properties for the Development of Solid Lipid Nanoparticles for Loading Lipophilic Substances

**DOI:** 10.1155/2021/6662867

**Published:** 2021-05-28

**Authors:** Putthiporn Khongkaew, Watcharaphong Chaemsawang

**Affiliations:** ^1^Department of Pharmacognosy and Pharmaceutical Chemistry, Faculty of Pharmaceutical Science, Burapha University, 169 Long-Hard Bangsaen Road, Saen Sook Sub-District, Mueang District, Chonburi 20131, Thailand; ^2^Department of Pharmaceutical Technology, Faculty of Pharmaceutical Science, Burapha University, 169 Long-Hard Bangsaen Road, Saen Sook Sub-District, Mueang District, Chonburi 20131, Thailand

## Abstract

Stingless bees are insects which are popularly bred by agriculturists in the eastern region of Thailand for the pollination of their orchards. The products from stingless bee breeding include bee honey and bee propolis. The objective of this experiment is to study the possibility of developing stingless bee propolis wax into solid lipid nanoparticles (SLN) by the comparison of five surfactants (Brij 721, Cremophor WO 7, Myrj 52, Poloxamer 188, and Tween 80). Each surfactant is used at three concentrations: 10%, 20%, and 30%. A master formula is selected according to the following: physical features, particle size, zeta potential, and entrapment. The results showed that Brij 721 and Myri 52 at 20% can be used in preparing SLN and have good preservation properties for vitamin E (size: 451.2 nm and 416.8 nm, zeta potential: - 24.0 and - 32.7; % EE: 92.32% and 92.00%, resp.). Therefore, they are further developed by adding the following drugs at low solubility: curcumin, ibuprofen, and astaxanthin. It is found that a formula using the surfactants Brij 721 and Myrj 52 at 20% have similar drug entrapment. The entrapment study involves curcumin 82%, ibuprofen 40%, and astaxanthin 67%. Moreover, the cytotoxicity test of blank solid lipid nanoparticle found no toxicty in fibroblast cell line (CRL-2522). Therefore, from this study, it is determined that stingless bee propolis wax has the potential to be developed to provide more efficient SLN in the future.

## 1. Introduction

Solid lipid nanoparticles (SLN) have been an interesting pharmaceutical delivery system since 1990. The system is composed of wax with a solid status at room temperature and body temperature, a surfactant, and water, creating lipid nanoparticles with features including a solid matrix [1]. There are important drugs spread throughout. In terms of popularity, particle size is mostly in the range of 50–500 nanometers [2–4]. In general, solid wax is used at 0.1–30%w/w in formulas. Wax with a melting point higher than 40°C is popularly used because the product will be stable at room temperature and not melt easily. Particularly, pharmaceutical products transported to tropical countries use such wax. More than one type of wax can be used, such as triglyceride, Glyceryltristearate (Dynasan®118), Glycerylbehenate (Compritol® 888 ATO), stearic acid, carnauba wax, or rice bran wax. However, the SLN is found to be problematic from the wax used, such as cases where the wax has a lower ability in active substance loading. There are also drugs pushed out from the system after storage for some time. Thus, studies to develop types of wax remain ongoing continuously. Stingless bees are insects that are simply bees without a sting, so they are popular for breeding to pollinate orchards, especially in the eastern region of Thailand. These bees are bred in large numbers and, apart from having no stinger, their habits for finding nectar are different from those of Indian hive bees or rock bees. That is, the stingless bee finds nectar not so far away, usually within 100–200 meters, and it swarms to flowers indiscriminately. The benefits of stingless bees are honey from the hive, beeswax, and propolis, which is a resinous substance gathered by stingless bees from natural sources such as broken bark, leaf buds, flower nectar, and pollen grain. Therefore, the color and composition of the wax are different according to the area. Generally, the components of propolis are different according to the source. Mostly, they include resin, vegetable balsam 50%, wax 30%, essential oil and aromatic oil 10%, pollen 5%, and other substances 5% [5–7]. Propolis gathered by stingless bees is mixed with saliva and beeswax, which makes its properties similar to those of aromatic glue, solid and brittle. When heated, it becomes sticky and soft. It is used by stingless bees for nesting and repairing their hives. Propolis is used for plugging broken nests. The study of propolis reveals that the substance from propolis extracted with alcohol provides pharmacological action, such as bacteriostatic effects and antioxidant activity.

Based on the previously mentioned information concerning the benefits of propolis, honey in the industry is separated from the honeycomb and the bee propolis is leftover from the industry. Still, villagers will use the remaining beeswax to make daily-use products, such as candles. Nonetheless, it is still not enough for the high amount of residual wax. In this research, a study was conducted to further develop residual wax for medicinal use to increase drug efficiency and reduce the amount of waste in industries, which is expected from the wax properties. It should be used as a replacement for synthesis wax in the future for the development of a drug delivery system.

## 2. Materials and Methods

Stingless bee propolis was collected from fruit gardens in Chanthaburi, Thailand. Ethanol, methanol, and hexane were purchased from Merck Millipore. Brij 721 was purchased from Croda. Poloxamer 188, Myrj 52, tocopherol acetate, and curcumin were purchased from Sigma Aldrich. Cremophor WO7, Tween 80, and ibuprofen were purchased from PC Drug and Chemical. Astaxanthin was kindly provided by Fuji Chemical. Dulbecco's Modified Eagle's Medium (DMEM) was purchased from Biowest. Fetal bovine serum (FBS), L-glutamax, and antibiotic/antimycotic were purchased from Invitrogen.

### 2.1. Stingless Bee Wax Extract

Stingless bee propolis was macerated in hexane for 24 hours, after which the extract was filtered with a sheet cloth. The extract was evaporated into concentrate by using a rotary evaporator machine. The wax was then evaporated using a water bath until all the hexane evaporated, followed by storing in a light brown bottle in a refrigerator at 4–8°C until use.

### 2.2. Characterization of Components of Stingless Bees Wax with GC-MS

The substance was dissolved in n-hexane (n-Hexane; Gas Chromatography MS SupraSolv® Grade, 1.00795 EMD Millipore, Merck). Then, the sample was filtered through a syringe filter (PTFE membrane, hydrophilic type, 0.45 *μ*m) before injection for analysis into GC-MS TRACE 1300, where it adhered to an automated liquid sampling set called a TriPlus RSH Autosampler and also to a tandem mass spectrometer called Triple Quadrupole Mass Spectrometer, model: TSQ 8000 Evo Thermo Scientific. The column used for chromatography was TR5-MS (5% phenyl methyl polysiloxane; 30 m × 0.25 mm ID, 0.25 *μ*m film thickness, Thermo Fisher Scientific). The amount of injection was 1 *μ*l split 1 : 10 temp injector of 300°C. For the temperature setting program of the column oven, the initial value was 80°C, which was maintained for two minutes, after which it was increased to 350°C at a speed of 5°C/minute and maintained for 10 minutes. The total time of analysis was 66 minutes per analysis. Carrier gas or helium was used under a flow rate of 1 mL/minute. For the conditions of mass spectrometry, mass ionization had an electron impact of 70 eV. The temperature of the transfer line used was 300°C, and the temperature of the ion source was 250°C. Data for the mass spectrum were collected in a full scan MS mode. The mass range was between 50 and 750 amu at a scan time (dwell time) of 0.2 seconds.

### 2.3. Characterization of Wax with FTIR and DSC

The four types of wax were as follows: (1) bee wax from the breeding stingless bee; (2) synthesized bee wax; (3) rice branch wax; (4) cholesterol. Uniqueness was analyzed with FTIR NICOLET 6700 and tested with Differential Scanning Calorimeters (DSC) (METTLER TOLEDO) by increasing the temperature by 10°C/minute using temperatures from 0°C to 400°C.

### 2.4. Preparation of Solid Lipid Nanoparticles Using Stingless Bee Wax

Each surfactant was used at three concentrations: 10%, 20%, and 30%. The surfactants were as follows: Brij 721, Myrj 52, Tween 80, Poloxamer 188, and Cremophor WO7.

In formula development, the first step of SLN development used tocopherol acetate (vitamin E) as a model drug. The preparation method was a microemulsion technique by mixing excipients, as in Table 1, followed by melting in a water bath. The water phase was poured into the oil phase and then blended with a homogenizer at a speed of 10,000 rpm for 30 minutes. After that, a microemulsion is immediately soaked in an ice bath to solidify. Then, proper formulas are selected to study the experiment in loading with other drugs, such as curcumin, ibuprofen, and astaxanthin.

### 2.5. Characteristics of Solid Lipid Nanoparticles

The particle size and surface charge are measured using a Nanosizer Series Zetasizer, while the characteristics were evaluated with a scanning electron microscope (SEM).

### 2.6. Entrapment Efficiency (%EE)

SLN formulation was centrifuged with ultracentrifuge (Amicon) MW 100 K at a speed of 10,000 rpm for 30 minutes and then washed with 50% ethanol and centrifuged two times to remove unloaded drugs. The particles on the membrane filter were dissolved with methanol to measure the absorbance by using UV spectrophotometry series U-2900UV at tocopherol acetate (vitamin E) with a wavelength of 295 nm. Curcumin was analyzed at a wavelength of 549 nm, ibuprofen at a wavelength of 264 nm, and astaxanthin at a wavelength of 470 nm. Curcumin, ibuprofen, and astaxanthin will be examined with the selected formulation from the information of tocopherol acetate.

### 2.7. Cell Lines and Cultures

The human fibroblast cells (CRL-2522) were purchased by our institution from American Type Culture Collection (ATCC). Cells were seeded in DMEM medium with 10% fetal bovine serum containing 1% antibiotic/antimycotic and cultured at 37°C in 5% CO2. The culture medium was changed every three days.

### 2.8. Cell Cytotoxicity

Cell cytotoxicity was assessed using the 3-(4, 5-dimethylthiazol-2-yl)-2, 5-diphenyltetrazolium bromide (MTT) assay. 100 *μ*l blank solid lipid nanoparticles prepared from Brij 721 or Myrj 52 were diluted in a cell culture complete medium containing 900 *μ*l. When 1 × 105 fibroblast cells had completed a 24-hour incubation in the 96-well plate, the culture medium was replaced with the particle solution and the cells were incubated for another 24 hours. After the completion of the 24-hour incubation, MTT at 5 mg/ml concentration was added at 10 *μ*l/well and incubated for another four hours at 37°C and 5% CO_2_, then the extract was drained and DMSO 100% 100 *μ*l/well was added to dissolve the formazan in intracellular. Finally, a microplate reader was used to measure at 570 nm and evaluate with an inverted microscope.

## 3. Results and Discussion

### 3.1. Characteristics of Stingless Bee Wax by Fourier Transform Infrared Spectroscopy (FTIR)

**Figure 1:**
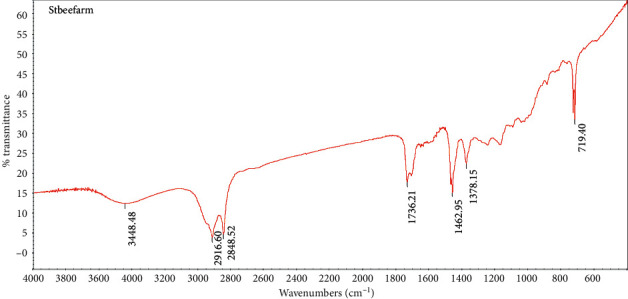
The spectrum represents the peak of stingless bees wax.

**Figure 2:**
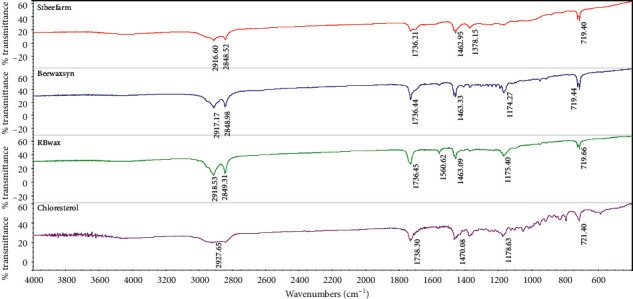
The FTIR spectra shows peaks of stingless bee wax (red), synthetic bee wax (blue), rice bran wax (green), and cholesterol (purple).

The FTIR spectrum of stingless bee wax is shown in Figure 1 and Table 2. Stingless bee wax has important points that consist of peaks at the point of 2800–2900, which is the sp3 C-H stretching peak of CH2 and CH3. This result represents the functional alkanes or alkyl (C-H) peak at the point of 1400, which is the sp3 C-H bending peak, while the peak shown at 1670–1780 is C=O stretching, which indicates a carbonyl functional group (C=O). The results show that stingless bee wax is a major component of fatty acids. Comparison with another type of wax in the FTIR spectrum in Figure 2 shows the consistency of the significant elements of stingless bee wax containing fatty acid as well as other types of wax because it establishes the same peak position, except that the position of the stingless bee wax at 1300 is not found in any other type of wax. It is found that there is a peak of C-O of ester in the fatty acid, which is an essential component of the stingless bee wax.

### 3.2. Characteristics of Stingless Bee Wax by Differential Scanning Calorimeters (DSC)

Differential Scanning Calorimeters (DSC) is used to measure the heat flow of the sample when being heated. The physical and chemical changes of the heated sample can be used for patterns of melting and the primary component.

**Figure 3:**
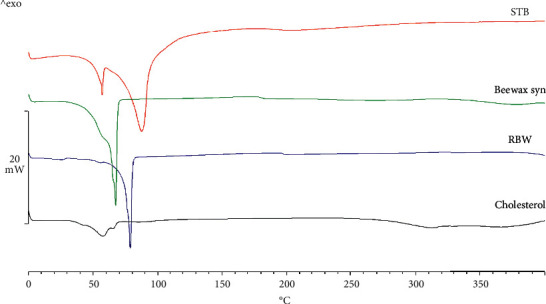
Thermogram of stingless bee wax, synthetic bee wax, rice bran wax, and cholesterol, respectively, by Differential Scanning Calorimeter.

From the results of the DSC thermogram (Figure 3) when compared with the peaks of synthetic bee wax and rice bran wax, there were different melting points compared with stingless bee wax, for which the proportional quantity of fatty acid is also different. Stingless bee wax has a higher melting point, which might be caused by the component of the fatty acids at saturated hydrocarbons [8]. This is an advantage for the lipid particle delivery system because of its superior stability compared with other kinds of wax in the part of the cholesterol, for which the main component is steroids [9, 10]. The thermal change is different from other substances.

### 3.3. Characteristics of Stingless Bee Wax by Gas Chromatography-Mass Spectrometry (GC-MS)

**Figure 4:**
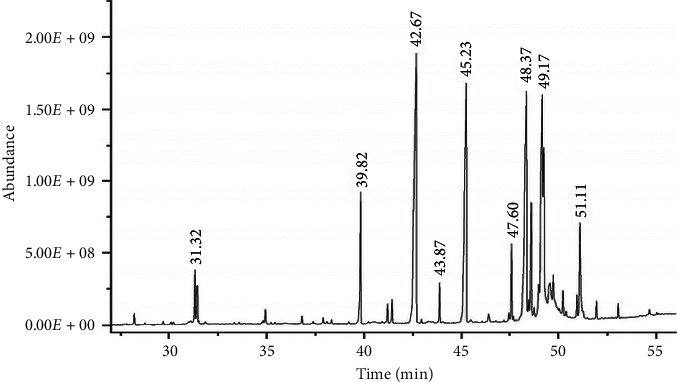
The GC-MS chromatrogram of stingless bee wax.

From the chromatogram of GC-MS (Figure 4), it was found that the major components of stingless bee wax included lipids, that is, long-chain fatty acids in the form of ester (3.24%), and hydrocarbons with a total length of 39.18%. Cholesterol and triterpenoids were also found from 48.37 minutes, which totaled 42.1% (Table 3). This conformed to the experiment by DSC, from which the thermogram showed that stingless bee wax had two melting point positions. To clarify, the melting point at 58.6°C was close to that of cholesterol. The other melting point at around 87-88°C was higher than the bee wax and rice bran wax, possibly from the components of the wax, which were mostly saturated hydrocarbons. For this reason, the melting point was higher than the lipids with unsaturated hydrocarbons. Therefore, the advantage of stingless bee wax when it is prepared as a solid lipid nanoparticle is that it has more stability than other natural lipids because it is not oxidized as easily. The arrangement of crystals was also lower than the unsaturated lipids. Moreover, the triterpenoids that were found conformed to the previous study, which discovered that propolis from natural bees had an antibacterial activity [10]. Substances found in stingless bee propolis *β*-amyrone, ester derivatives, and 9, 19-cyclo-9*β*-lanost-24-en-3*β*-ol were found to have antioxidation and antibacterial properties [11–13] Hence, applying prepared solid lipid nanoparticles for medical or cosmetic use would tend to be beneficial.

### 3.4. Characteristics of Stingless Bee Wax Solid Nanoparticles

**Figure 5:**
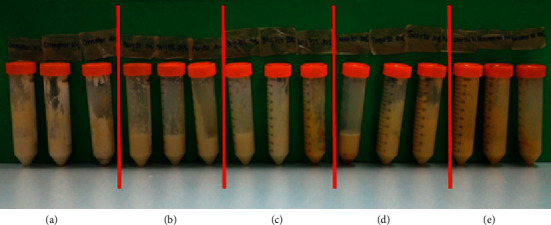
Physical properties of each surfactant. (a) Cremophor WO7, (b) Myrj 52, (c) Brij 721, (d) Tween 80, and (e) Poloxamer 188 (surfactant concentration 10%, 20%, and 30%).

**Figure 6:**
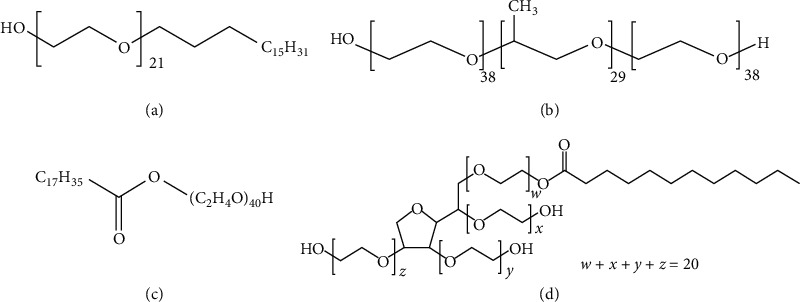
Chemical structure of each surfactant. (a) Brij 721, (b) Poloxamer 188, (c) Myrj 52, and (d) Tween 80.

**Figure 7:**
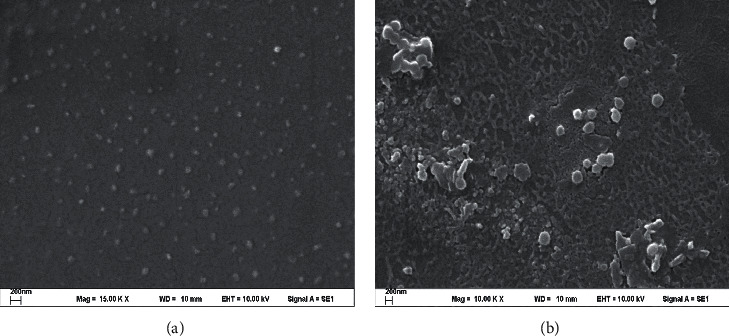
The photographs of the scanning electron microscope. (a) Brij 721, 20%; (b) Myrj 52, 20%.

From the preparation of the SLN particles with five types of surfactants, it was found that Cremophor WO 7 could not prepare SLN. The cream was very thick and unable to separate the particles, thus resulting in the inability to collect any particles for the physical quality assessment. Regarding the other surfactants, Brij 721, Myrj 52, Tween 80, and Poloxamer 188 (Table 4 and Figure 5), they showed similar results in terms of type and concentration per particle size. At a 10% concentration, the particle size is smaller. The ability of surfactant to reduce surface tension is not yet complete, thus creating particles that are very small to very large. Moreover, the entrapment of vitamin E is incomplete. For instance, 10% of Poloxamer 188 has lower entrapment efficiency than using concentrations of 20% and 30%. As such, this represented incomplete particle formation that affected less drug entrapment. However, when the concentration of the surfactants was increased, the particle size became slightly larger, even though the particle result was improved due to the reduction of surface tension and the higher entrapment efficiency. However, the particle size increased when using nonionic surfactants in the concentration range. The research of Hazzah et al. [14] can explain the phenomenon that occurs when increasing the concentration of the surfactant, where the particle size would be larger. The force of the hydrocarbon chain of the surfactant attracted Van Der Waals forces among the hydroxyl group in the hydrocarbon chain, causing the wax droplets to coalesce into large droplets before solidifying. Alternatively, the particle would become smaller again when the concentration of the surfactant increased. The mechanism of the reduced surface tension is strong enough to cover the thermodynamic force. Therefore, the particles can remain in their shape, even with a high surface area, resulting in smaller and more stable particles [15]. Although the high concentration of the surface tension minimized the particles, the percentage of the entrapment was also reduced. The decrease in entrapment is because of the very high concentration of surfactants as solubilizers; hydrophobic agents can be soluble with micellar mechanisms. Therefore, some drugs dissolve in the surfactant, such as micelle, and disperse in the aqueous phase, but do not dissolve in the oil phase [16, 17]. A drop of wax is created when solidified into particles with lower drug content than it should be. From the experimental results of the vitamin E loading SLN preparation (Table 5), a suitable concentration of the surfactant was 20% with the maximum percentage of entrapment, when considering the parameters and physical characteristics, as previously mentioned. Regarding the type of surfactants, Brij 721 and Myrj 52 offered the best particle properties with a smaller size and higher entrapment. It was also found that Brij 721 and Myrj 52 had a linear molecule structure, and the number of carbon chains was lower than Tween 80, and Poloxamer 188 (Figure 6). Tween 80 had a long carbon side chain, which had a branch chain type, while Poloxamer 188 also had a linear polymer, but the molecular weight was higher. Hence, the structure is bent into the U shape when poloxamer 188 forms the micelles, with the hydrophilic part facing outwards [18]. This was the reason why the molecules of Brij 721 and Myrj 52 were small and could cover the particle's surface with more stability. Concerning microemulsion preparation using the surfactant with small particles or cosurfactant, the microemulsion remained more stable than using the surfactant with large particles [15, 19–22].  Figure 7 shows the morphology of SLN from both types of surfactants (Brij 721; Myrj 52), which had spherically-shaped particles under SEM. For the results of zeta potential values, nanometer particle size typically requires a charge of −30 or +30 mv [23–25] and above to maintain particle stability. Zeta potential values closer to zero will result in particle aggregation. The findings showed a negative charge of the particles due to the carboxylic group in the fatty acid found in wax. The results found that nonionic surfactant will not significantly affect the value of zeta potential.

The research results show that the formulations prepared from Brij 721, 20%, had an average smaller particle size than Myrj 52, 20%, but the findings were similar when evaluating the % entrapment efficiency (Table 4). The results show that curcumin has the highest drug entrapment because of its high molecular weight; the higher wax solubility creates a better possibility for wax solubility [26]. In addition, ibuprofen has less entrapment efficiency because its chemical structures have polar functional groups such as carboxylic groups. Therefore, the drug may be easily pushed out of liquid wax during wax solidification [27, 28].

### 3.5. Cytotoxicity

**Figure 8:**
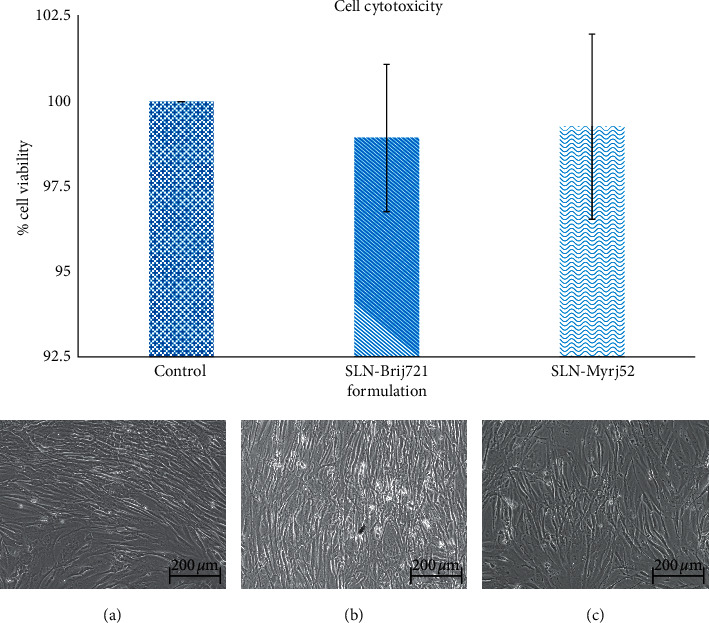
Cell cytotoxicity. (a) Control, (b) blank SLN from Brij 721, and (c) blank SLN from Myrj 52.

The cytotoxicity of SLN from stingless bee propolis was determined using the MTT assay method on the fibroblast cell line. Data showed both SLN prepared from Brij 721 and Myrj 52 had no significant difference in toxicity when compared with the control group or the preparation from Brij and Myrj (Figure 8). Observation under a microscope to determine particles cannot be done because the size of SLN is too small. Furthermore, another research has also found that the SLN delivery system is safe for human cells. It also found that fibroblast cells can deliver SLN into a cell by endocytosis pathway before degrading with lysozyme to release drugs [29, 30]. This might also be the reason why the SLN particles are invisible outside the cells under a microscope. The previous research by Karting et al. [31], concerning delivery of glucocorticoid by SLN, shows that SLN can increase the permeation of glucocorticoids by three times compared with the control. Furthermore, the survival rate of the cells is 94.5% after 18 hours of incubation. The results conformed to this research. Moreover, the SLN system reduced skin irritation that had the irritation quality in the animal test [32]. From the previous research, it can be concluded that the SLN delivery system is safe and could be developed for use on the human body. Furthermore, it increases the chance of the absorption rate and decreases the toxins from the medicine. This observation provides support for the development of solid lipid nanoparticles from stingless bee propolis for future pharmaceutical purposes.

## 4. Conclusion

According to the research results, it was found that the wax of stingless bees could be developed and used as a medicine delivery system. The study found that the components in stainless bee wax include fatty acid and terpenoids. The terpenoids were reported for their antimicrobial activity. Therefore, it could be studied or developed further for pharmaceutical use in the treatment of localized infections. In addition, the physicochemical test from DSC found that stingless bee wax had high physicochemical stability compared with other types of wax, resulting in more stability under long-term storage. Lastly, the toxicity test showed no toxicity in the fibroblast cells, in the prepared formula from either Brij 721 or Myrj 52. Thus, the delivery system of a solid lipid nanoparticle could be used in real situations in the future, by either transdermal drug delivery or oral medication. However, this research study is only a basic evaluation of the characteristics of stingless bee wax. Further studies must be performed in greater depth to assess its stability and development of the delivery system.

## Figures and Tables

**Table 1:** Formulation SLN.

	Rx10% surfactant (g)	Rx20% surfactant (g)	Rx30% surfactant (g)
Stingless bee wax	10	10	10
Ethyl alcohol	14	14	14
API	1	1	1
Surfactants	10	20	30
Water qs. to	100	100	100

**Table 2:** FTIR characteristic peaks of stingless bee wax.

Wave number^−1^	Appearance	Group
1462.95	Weak	C-H bending
1736.21	Strong	C=O stretching
2000–2500	Weak	Overtone of aromatic compound
2916.60, 2848.52	Strong	C-H stretching
3448.48	Strong	O-H stretching

**Table 3:** Tentative compounds found in stingless bee wax.

*tR* (min)	Compound	Relative abundance (%)
30.12–31.86	Fatty acid ester	3.24
	Linoleic acid, methyl ester	
	Linolenic acid, methyl ester	
	*cis*-9, *cis*-12-linoleic acid	
	Linoleic acid, ethyl ester	
	Oleic acid, ethyl ester	
	Elaidic acid, ethyl ester	
	Stearic acid, ethyl ester	
39.82	Hepta cosine	4.01
42.67	Nonacosane	20.01
45.23	Hentriacontane	15.16
48.37	Lupen-3-one	15.12
48.62	*β*-Amyrone	3.68
49.17	9, 19-Cyclo-9*β*-lanost-24-en-3*β*-ol	11.36
49.26	*β*-Amyrin 3-acetate	7.83
51.11	23-(Phenylsulfanyl) lanosta-8, 24-dien-3-ol	4.11
	Others	6.31
	Total unknown	9.17

**Table 4:** The comparison of the particle size of zeta potential and % entrapment efficiency of the formation containing vitamin E (*n* = 3); ZP: zeta potential.

Surfactant (%)	Size	ZP	% EE
*Brij 721*			
10	461.4 ± 17.82	−26.0 ± 1.36	96.62 ± 0.00
20	451.2 ± 6.58	−24.0 ± 0.51	92.32 ± 0.19
30	292.1 ± 23.60	−13.3 ± 0.45	74.59 ± 0.13

*Cremophor WO 7*			
10	N/A	N/A	N/A
20	N/A	N/A	N/A
30	N/A	N/A	N/A

*Myrj 52*			
10	366.4 ± 7.73	−28.4 ± 0.41	91.48 ± 0.09
20	416.8 ± 44.61	−32.7 ± 0.96	92.00 ± 0.00
30	366.8 ± 3.09	−27.5 ± 1.12	80.86 ± 0.00

*Poloxamer 188*			
10	466.9 ± 64.20	−30.6 ± 1.13	53.92 ± 0.07
20	759.3 ± 88.52	−32.9 ± 0.15	73.64 ± 0.11
30	388.0 ± 1.99	−32.2 ± 0.35	74.23 ± 0.12

*Tween 80*			
10	357.8 ± 39.97	−27.3 ± 2.72	63.95 ± 0.00
20	756.6 ± 61.24	−24.1 ± 0.45	67.10 ± 0.00
30	312.2 ± 17.11	−25.2 ± 3.11	81.04 ± 0.00

**Table 5:** The comparison of the particles of zeta potential and % entrapment efficiency (*n* = 3).

	Size	ZP	% EE
*Curcumin*			
Brij 721, 20%	426.3 ± 6.93	−28.0 ± 0.42	92.37 ± 0.00
Myrj 52, 20%	445.5 ± 14.31	−31.5 ± 0.49	74.32 ± 0.14

*Ibuprofen*			
Brij 721, 20%	443.3 ± 6.93	−19.9 ± 0.42	40.68 ± 0.00
Myrj 52, 20%	442.2 ± 14.31	−37.8 ± 0.49	40.97 ± 0.14

*Astaxanthin*			
Brij 721, 20%	407.5 ± 6.93	−25.7 ± 0.42	65.33 ± 0.00
Myrj 52, 20%	451.3 ± 14.31	−33.6 ± 0.49	68.20 ± 0.14

## Data Availability

Data used in this study are available upon request.
